# Impact of HPV Infection on the Immune System in Oropharyngeal and Non-Oropharyngeal Squamous Cell Carcinoma: A Systematic Review

**DOI:** 10.3390/cells8091061

**Published:** 2019-09-10

**Authors:** Jerome R. Lechien, Imelda Seminerio, Géraldine Descamps, Quentin Mat, Francois Mouawad, Stéphane Hans, Morbize Julieron, Didier Dequanter, Thibault Vanderhaegen, Fabrice Journe, Sven Saussez

**Affiliations:** 1 Department of Otolaryngology and Head and Neck Surgery, CHU Saint-Pierre, 1000 Brussels, Belgium; 2 Laboratory of Human Anatomy and Experimental Oncology, Faculty of Medicine, Research Institute for Health Sciences and Technology, University of Mons (UMONS), Avenue du Champ de Mars, 8, B7000 Mons, Belgium; 3 Department of Otolaryngology and Head and Neck Surgery, Centre Oscar Lambret, 59000 Lille, France; 4 Department of Otolaryngology and Head and Neck Surgery, CHU de Charleroi, 6042 Charleroi, Belgium; 5 Department of Otolaryngology and Head and Neck Surgery, CHU de Lille, Université Lille 2, 59000 Lille, France; 6 Department of Otolaryngology and Head and Neck Surgery, Foch Hospital, 92150 Paris, France; 7 Laboratory of Oncology and Experimental Surgery, Institut Jules Bordet, Université Libre de Bruxelles, Rue Heger-Bordet, 1, B1000 Brussels, Belgium

**Keywords:** HPV, cancer, head, neck, immune, cells

## Abstract

Objectives: To review the current knowledge regarding the involvement of human papilloma virus (HPV) infection and the immune system in the development of head and neck squamous cell carcinoma (HNSCC). Methods: An electronic literature search was conducted to identify articles published between 1990 and 2019 pertaining to tumor-infiltrating immune cells (TICs) in HNSCC using the Preferred Reporting Items for Systematic Reviews and Meta-Analyses guidelines. Issues of clinical relevance, including tumor location, the number of tumor samples, the inclusion of additional specimens (dysplastic or normal mucosa), tumor size, methods used for HPV detection, relationship between antigen expression and patient characteristics (age, gender, smoking, alcohol consumption, etc.), and prognostic data (overall survival (OS) and recurrence-free survival (RFS)) were assessed by four blinded investigators. Results: The search identified 335 relevant studies, of which 41 met the inclusion criteria. Of these, 7 studies focused on the peripheral blood immune cell concentration in patients with HNSCC according to HPV status, and 36 studies investigated TICs in the intraepithelial and/or stromal compartment(s) according to HPV status. The immune cells studied were CD8+ T cells (N = 19), CD4+ T cells (N = 7), regulatory T cells (Tregs, N = 15), macrophages (N = 13), myeloid-derived suppressor cells (MDSCs, N = 4), and Langerhans cells (LCs, N = 2). Conclusions: Irrespective of tumor location, CD8+ and CD4+ T cells appear to play a key role in the development of HPV−related HNSCC, and their infiltration is likely associated with a significant impact on OS and RFS. To date, the roles and prognostic value of Tregs, macrophages, DCs and MDSCs remain unclear.

## 1. Introduction

Head and neck squamous cell carcinoma (HNSCC) is the sixth most common cancer in men and the eighth most common cancer in women, accounting for over 600,000 new cases per year worldwide [1,2]. The main risk factors for HNSCC are alcohol and tobacco consumption and persistent infection with human papillomavirus (HPV), which is associated with the rising incidence of oropharyngeal cancer in the United States and Europe [3,4]. HPV infection is predominantly attributable to subtypes HPV−16 and HPV−18, but geographical heterogeneity has been reported between continents [5]. Nondrinking and nonsmoking patients with HPV−induced HNSCC often present with advanced cancer, but they have a better prognosis than patients with HNSCC associated with smoking and drinking [6,7,8,9,10,11]. The better prognosis of patients with HPV−induced HNSCC could be due to the interactions between HPV antigens and the host-immune system and a better immune response against these tumors. In this respect, many studies have demonstrated significant differences in the composition of tumor-infiltrating immune cells (TICs) in HPV−induced and non-HPV−induced HNSCC over the past two decades [12,13]. Moreover, the results of some of these studies indicate that the overrepresentation of some immune cells should be considered a significant prognostic factor for HNSCC patients [14,15]. However, to date, there has been no systematic review summarizing the current knowledge of TICs and the tumor-host interaction in HPV−induced HNSCC.

The aim of this systematic review is to discuss the current knowledge of the involvement of HPV infection and the immune system in the development of HNSCC.

## 2. Materials and Methods

The criteria for considering studies for the systematic review were based on the population, intervention, comparison, and outcome (PICO) framework [16].

Types of studies: Prospective or retrospective clinical trials published in peer-reviewed journals were included in this review. Studies were included if they explored TICs in HNSCC, including oropharyngeal, laryngeal, hypopharyngeal and oral squamous cell carcinoma. We included studies published in English and French.

Participants and inclusion/exclusion criteria: Papers were included in the analysis if they clearly reported TICs in excised HNSCC samples or pretherapeutic biopsies from patients who were treated with conventional treatment (i.e., surgery, chemoradiation, chemotherapy, or immunotherapy). Papers that examined the recruitment of immune cells through blood analyses were also included to improve the understanding of the tumor immune microenvironment. HPV detection was required to have been performed through DNA or p16 analysis of the tumor samples. Studies focused on non-HNSCC were excluded.

Outcomes: The first outcome was the study of TICs in the intraepithelial (or intratumoral) and stromal compartments in HNSCC according to HPV status. To better understand the involvement of the immune system in the development of HPV−induced HNSCC, investigations into the expression of both cytokines and checkpoint proteins involved in tumor development were performed. Table 1 summarizes the immune cells included in this systematic review [17,18,19,20,21,22,23,24,25]. The second outcome was the study of tumor characteristics (tumor size, node, metastasis, and histopathology) and/or prognostic data (overall survival (OS) and recurrence-free survival (RFS)) in relation to TICs and HPV status.

Intervention and comparison: In the case of the study of the prognostic value of TIC, the authors were required to have treated their patients with conventional surgical or conservative treatments.

### 2.1. Search Strategy

Four authors (JRL, IS, TV, and QM) conducted searches in PubMed, Cochrane, and Scopus databases to identify articles published between January 1990 and June 2019 pertaining to the relationship between HPV infection and TICs in the development of HNSCC. Clinical studies were screened if they had database abstracts, available full texts or titles that referred to these conditions. The following keywords were used: ‘HPV’, ‘cancer’, ‘carcinoma’, ‘head’, ‘neck’, ‘immune’, and ‘cell’. Final article selection was determined by the four authors, who provided a critical analysis of the publication content. The review was conducted according to the PRISMA checklist [26]. Institutional review board approval was not required.

### 2.2. Epidemiological Characteristics and Outcomes

An analysis of the locations of tumors, the number of tumor samples, the inclusion of additional specimens (i.e., dysplastic tissues or normal mucosa), tumor size, the relationship between antigen expression and patient characteristics (age, gender, smoking, and alcohol consumption), and prognostic data (OS and RFS) was performed. The method used for HPV detection was carefully analyzed to provide a methodologically critical analysis of the included studies.

## 3. Results

### 3.1. Study Characteristics

**Figure 1:**
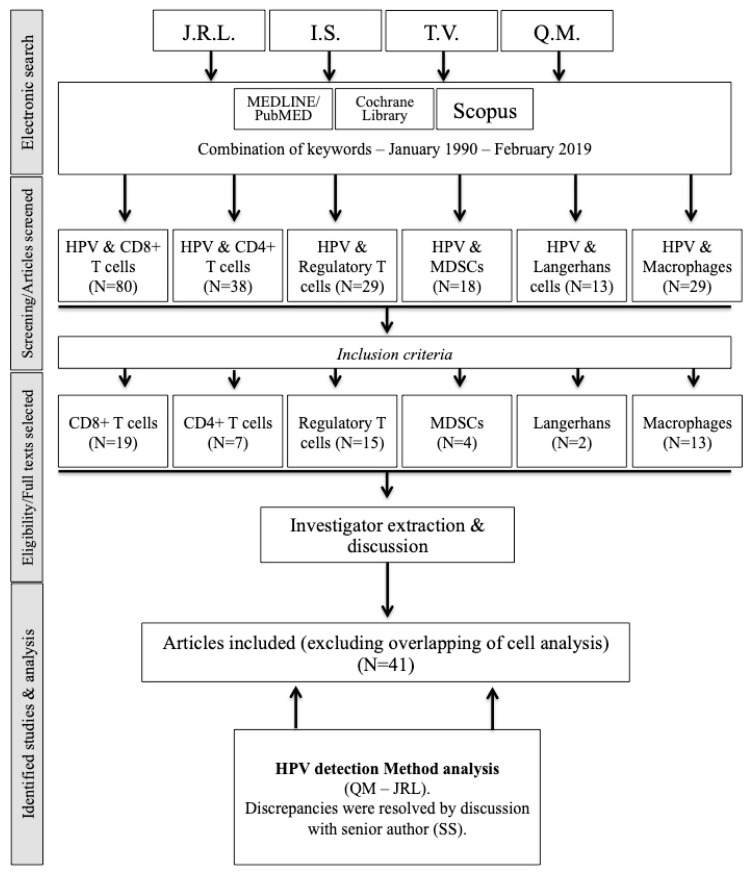
Flow chart. Abbreviations: HPV = human papilloma virus; MDSC = myeloid-derived suppressor cells.

The initial screening identified 335 studies, of which 41 met our inclusion criteria (Figure 1). A total of 7 studies investigated the peripheral blood immune cell concentration in patients with HNSCC according to HPV status (Table 2) [27,28,29,30,31,32,33]. Three studies focused precisely on the evolution of the peripheral blood immune cell concentration between pre- and posttreatment (surgery or chemoradiation) [28,29,31].

A total of 36 studies examined TICs in the intraepithelial and/or stromal compartment(s) according to HPV status (Figure 1). Of the papers that investigated several different immune cell categories, some focused on oropharyngeal SCCs, whereas others focused on HNSCCs, including oropharyngeal and other anatomical localizations. The studies are shown in Table 3, Table 4, Table 5 and Table 6 [12,15,24,30,31,32,33,34,35,36,37,38,39,40,41,42,43,44,45,46,47,48,49,50,51,52,53,54,55,56,57,58,59,60,61,62,63,64].

### 3.2. Oropharyngeal SCCs

A total of 10 and 5 studies addressed CD8+ and CD4+ T lymphocytes (Table 3) [34,36,37,38,40,41,43,45,47,51]. Seven studies focused on regulatory T cells (Tregs) (Table 4) [34,36,38,43,45,53,54], and 5 focused on macrophages (Table 5) [34,56,58,59,60]. No studies addressed myeloid-derived suppressor cells (MDSCs) or Langerhans cells (LCs).

### 3.3. HNSCCs

A total of 8 and 2 studies addressed CD8+ and CD4+ T lymphocytes (Table 3) [35,39,42,44,46,48,49,50,52]. Eight studies focused on regulatory T cells (Tregs) (Table 4) [12,15,24,33,35,39,42,49], 8 focused on macrophages (Table 5) [14,39,44,49,55,57,61,62], and 6 focused on myeloid-derived suppressor cells (MDSCs) or Langerhans cells (LCs) [32,39,44,57,63,64].

### 3.4. HPV and the Peripheral Blood Concentrations of Immune Cells

Because some immune cells are derived from bone marrow and are transported to tissues through the circulation, the peripheral blood number of immune cells (PBNI) was explored in 7 studies. Overall, it seems that patients with HNSCC have a higher PBNI than healthy individuals [28,29,32,33]. The role of HPV status is still unclear because only one study reported significant differences between the PBNI in patients with HPV+ and HPV− HNSCC [30]. Two authors found a high concentration of circulating CD8+ T cells against the E6 and E7 proteins in the majority of HPV+ patients with oropharyngeal SCC [29,31]. However, these observations were not supported by Heusinkveld et al., who included patients with oropharyngeal and non-oropharyngeal SCC [27]. According to Lukesova et al., natural killer cells are another type of cell that can be increased in patients with HPV+ oral or oropharyngeal SCC [30]. The other types of immune cells were less well studied, and it is difficult to pursue some lines of investigation.

According to two studies [28,29], the treatment type could have an impact on the PBNI, but the differences between these two studies (in terms of tumor location, types of treatment, and HPV detection methods) limited our comparison. The relationship between PBNI and OS was addressed in two studies [30,31]. Masterson et al. found that a high blood concentration of CD8+ T cells enhanced immunoreactivity to antigen E7, which was associated with improved OS in patients with oropharyngeal SCC [31]. In the same vein, Lukesova et al. demonstrated that patients with oral or oropharyngeal HPV+ SCC and a high Treg blood concentration had improved OS and RFS [30].

### 3.5. HPV and CD8+/CD4+ T Cell Tumor Infiltration

A total of 19 studies addressed CD8+ T cell infiltration (Table 3). Among the papers focusing on oropharyngeal SCC, the majority reported higher stromal or intraepithelial CD8+ T cell infiltration in HPV+ than in HPV− SCC [36,37,38,40,43,45,46,47,51]. Only Wansom et al. did not find significant differences between these two types [34]. These results are relatively similar to those of studies that investigated HNSCC. Russel et al. and Badoual et al. observed increased CD8+ T cell infiltration in the intraepithelial compartment in HPV+ HNSCC [35,39], whereas when investigating laryngeal, oral, hypo- and oropharyngeal tumor samples, Balermpas et al. did not find significant differences between HPV+ and HPV− SCC [42]. In a second study, the same authors reported the occurrence of different patterns of CD8+ T cell infiltration according to the tumor location; CD8+ T cell infiltration was higher in HPV+ than in HPV− tumor samples only in oropharyngeal SCC [46]. Other authors reported an increase in infiltration in HPV+ HNSCC irrespective of the compartment [44,48,49,50].

The high CD8+ T cell infiltration in HPV+ tumors was associated with the high expression of checkpoint proteins, including PD-1 [44,48,52], LAG-3 [48], and Tim-3 [35], in HNSCC. The expression of PD-1 by HPV+ tumors was reported in the study by Kansy et al., who found a significant association between the CD8+ antigens CD8A and CD8B and the expression of PD-1 [52]. Partlova et al. also found that HPV+ tumors had higher expression of PD-1 mRNA than did HPV− HNSCC tumors [44]. The activity of CD8+ T cells is mediated by many cytokines, leading some authors to study the expression profile of TICs. Partlova et al. found that TICs in HPV+ SCC comprised IFN-g+ and IL-17+ CD8+ T cells [44], supporting the key role of these cytokines in the inflammatory reaction related to HPV. Overall, a high number of TICs (including CD8+ T cells) in HPV+ tumors was associated with the increased secretion of the following proinflammatory cytokines: CCL-17, CCL-21, IL-2, IL-4, IL-8, IL-10, IL-12, IL-17, IL-21, TNF-a, and IFN-g [39,44]. However, neither of these two studies demonstrated that cytokine expression was directly related to the level of CD8+ T cells.

A high level of CD8+ T cell infiltration was associated with improved OS and RFS in all studies focusing on HPV+ oropharyngeal SCC [34,36,37,40,41,51]. Moreover, Ward et al. found that patients with HPV+ oropharyngeal SCC with low levels of tumor-infiltrating lymphocytes had the same prognosis as those with HPV− oropharyngeal SCC [41]. Similar overall findings were observed in HNSCC for OS [42,46,48,50] and RFS [46,48,50]. One study reported that low CD8+ infiltration was associated with a high risk of metastases [46].

### 3.6. HPV and CD4+ T Cell Tumor Infiltration

Seven publications have reported findings regarding CD4+ T cell infiltration in HPV−induced SCC (Table 3). Overall, CD4+ T cell stromal infiltration was reported to be higher in HPV+ than HPV− oropharyngeal SCC [37,38,40,43]. In HNSCC, Balermpas et al. reported similar levels of CD4+ T cell infiltration in HPV+ and HPV− HNSCC [42]. Regarding the prognostic value of CD4+ T cell infiltration, Nordfors et al. did not find a significant association between the level of CD4+ infiltration and OS in patients with HPV+ and HPV− oropharyngeal SCC [40]. Similar results were found in the study by Balermpas et al., in which CD4+ expression was not associated with a good prognosis [42]. Regarding the subpopulation of CD4+ T cells, Krupar et al. studied the infiltration of Th17 cells in oropharyngeal SCCs. They found that there was a significantly lower percentage of Th17+ T cells in the intratumoral compartment of HPV+ patients.

### 3.7. Regulatory T Cell Infiltration

Fifteen studies have examined the tumor infiltration of Foxp3 Tregs according to HPV status, including 7 that were focused on oropharyngeal SCC (Figure 1, Table 4). Overall, a few studies reported that the degree of Foxp3 Treg infiltration did not differ according to HPV status in oropharyngeal SCC [34,45], but the majority of the authors observed increased infiltration in HPV+ oropharyngeal SCC, especially in the intratumoral compartment [36,38,43,53,54]. When analyzing HNSCC studies, Kindt et al. demonstrated that Foxp3 Treg infiltration increases with tumor progression; this increase is more important in HPV+ patients [24].

Four studies reported increased infiltration of Foxp3 T cells in the intratumoral [24,35,49] or stromal [39] compartment in HPV+ compared with HPV− HNSCC. Additionally, a study by Partlova et al. reported a slightly lower proportion of Treg cells in HPV+ HNSCC. These authors also found higher levels of cytokines and chemokines in HPV+ patients than in HPV− patients, but they did not report which cells produced these cytokines [44]. In 3 studies, the authors found that the infiltration of Foxp3 T cells was similar in HPV+ and HPV− SCC [15,33,42]. Two studies aimed to study the association between Treg infiltration and checkpoint protein expression [33,35]. Irrespective of HPV status, Badoual et al. and Lechner et al. found that the infiltration of Tregs was associated with higher levels of checkpoint protein expression (i.e., PD-1) in HNSCCs [33,35].

Regarding the prognostic value of Treg infiltration, the high infiltration of Foxp3 Tregs was associated with improved OS or RFS in both oropharyngeal cancer [34,36,53,54] and HNSCC [15,24,35,39]. Moreover, the results of the study by Kindt et al. suggested that the number of stromal Tregs is a strong prognostic factor that is independent of other risk factors, including tobacco and alcohol consumption and HPV status [24]. To date, only Balermpas et al. did not find a significant association between the degree of Treg infiltration and OS [42].

### 3.8. Macrophage Infiltration

From our literature search, we identified 5 and 8 publications that investigated macrophage infiltration in oropharyngeal and HNSCC according to HPV status, respectively (Figure 1 and Table 5). In 3 studies, CD68+ macrophages showed increased infiltration in HPV+ compared to HPV− oropharyngeal SCC [56,57,58,59]; only one publication did not corroborate this finding [34]. In both oropharyngeal and nonoropharyngeal SCC, CD68+ macrophage infiltration increases between dysplastic tissues and carcinoma; the macrophage infiltration density is higher in the advanced stages [14,57]. Yu et al. focused on the CD68+/CD163+ macrophage (M2) subpopulation in oral SCCs, and they did not find a significant difference between HPV+ and HPV− oral SCC according to tumor progression and HPV status [57]. However, the relationship between CD68+ macrophage infiltration and HPV status is more controversial in HNSCC. Indeed, 5 authors did not find a significant difference between HPV+ and HPV− HNSCC regarding CD68+ macrophage infiltration [39,44,55,57,62], while macrophage infiltration increased in the intratumoral compartment in HPV+ HNSCC in 3 studies [14,49,61]. Among these studies, Ou et al. reported a tendency toward a higher proportion of M2 macrophages in HPV− HNSCC [61].

The relationship between CD68+ macrophage infiltration and checkpoint protein expression has been examined in two studies [56,59]. On the one hand, Lyfor-Pike et al. reported that CD68+ macrophages expressed high levels of PD-1 [56]. On the other hand, Oguejiofor et al. found that HPV− oropharyngeal SCC had an increase in CD68+ PD-L1+ macrophages compared to HPV+ tumors; these tumors were associated with improved OS compared to HPV− oropharyngeal SCC with low CD68+ PD-L1+ infiltration [59]. Lee et al. found that the high infiltration of CD68+ macrophages was associated with poor OS and DSS [58], while Seminerio et al. demonstrated that the high intratumoral infiltration of CD68+ macrophages was linked with shorter OS in patients with HNSCC [14]. Finally, Welters et al. found that the increase in dendritic cell-like macrophage infiltration in HPV+ oropharyngeal SCC was correlated with improved OS and a low risk of lymph node metastases [60].

### 3.9. Dendritic Cell Infiltration

#### 3.9.1. Myeloid-Derived Suppressor Cells

A total of 4 included studies evaluated the relationship between MDSC infiltration and HPV status in HNSCCs using different antigens, including CD11b, Arg-1, INOS, and STAT3 (Figure 1, Table 6). MDSC infiltration increased throughout tumor progression [32,39], and overall, no significant difference was found between HPV+ and HPV− HNSCC [32,39,57]. Moreover, MDSC infiltration seemed to be higher in advanced-stage than in early-stage HNSCC [32].

Regarding the expression of checkpoint proteins, Yu et al. found a positive correlation between the infiltration of MDSCs and PD-L1 expression [57]. For CD8+ T cells, Partlova et al. reported high proinflammatory cytokine expression in tumor samples, characterized by a high infiltration of MDSCs, but they did not demonstrate a potential association [44]. To date, no study has investigated the prognostic value of MDSC infiltration according to HPV status.

#### 3.9.2. Langerhans Cells

Only 2 studies assessed LC infiltration according to HPV status (Table 6). In a cohort of 27 patients with oral SCC, Perreira et al. did not find a significant difference between HPV+ and HPV− oral SCC [63]. More recently, Kindt et al. observed that LC infiltration increased in HNSCC throughout tumor progression but decreased in the presence of HPV infection. Moreover, there was a significant association between the LC infiltration level and cT and tumor node status [24]. The LC infiltration level was positively associated with improved OS and RFS in HPV− but not HPV+ HNSCC.

### 3.10. SCC Location and the Detection of HPV

The majority of studies (N = 17) were exclusively performed on oropharyngeal SCC [28,29,31,34,36,37,38,40,43,45,47,53,54,56,58,59]; 2 focused on oral SCC [57,62], 1 focused on nodes with unknown primary SCC [51], and the rest included upper aerodigestive tract SCC in different locations. TICs were compared with dysplastic and normal mucosa samples in 10 studies [15,24,32,33,39,44,52,56,57,64].

To detect HPV infection in tumor samples, 4 authors exclusively assessed p16 expression in samples [42,48,53,61], while 36 authors performed DNA detection using different forms of PCR (in situ hybridization, E7 RT-PCR, E6/E7 multiplex qPCR, etc.). In one study, the detection method was not specified [34].

## 4. Discussion and Perspectives

This systematic review emphasizes many lines of evidence and reveals uncertainties regarding the role of immune cells in the development of HPV−induced HNSCC. These findings are summarized in Table 7. Our analysis indicates that in both oropharyngeal and nonoropharyngeal SCC, HPV infection is associated with increased CD8+ T cell infiltration and PD-1 expression by CD8+ T cells and improved OS. CD8+ T cells interact with their environment through multiple cytokines, especially IFN-g and IL-2, -4, -8, -12 and -17 [39,44]. The increased expression of IL-17 in HPV+ HNSCC cells is associated with the infiltration of Th17 lymphocytes, resulting from the differentiation of CD4+ T cells. Thus, Krupar et al. reported an increased number of Th17 lymphocytes in TICs in HPV+ oropharyngeal SCC [43]. This observation makes particular sense according to studies that demonstrated increased CD4+ T cell infiltration in HPV+ SCC [37,38,40,43]. Because recent studies have suggested that lymphocyte plasticity can occur during HNSCC development, which is characterized by Th1 phenotype expression in Th17 cells [65,66], these findings could support a potential relationship between CD8+ and CD4+ T cells in HPV−induced SCC. CD4+ T cells could be converted into Th17 cells, potentiating the cytotoxic effects of CD8+ T cells against HPV−induced SCC antigens. According to several studies, the development of TICs, including CD8+ T cells, is associated with an increase in the detectable PBNI [28,29,32,33]. In this respect, and considering the data of Parikh et al., it is probable that the detection of CD8+ T cells against E6 or E7 HPV proteins could be used in future studies to better characterize tumor immunogenicity and, as recently suggested, the response to some treatments (i.e., immunotherapy and chemoradiation) [29,31]. However, scholars should remain prudent because the usual method of assessing the PBNI (blood sampling) is performed at a single time point and does not usually consider the variation in the blood concentrations of immune cells due to other factors related to the circadian rhythm or other external causes.

Macrophages have also been extensively studied in relation to HPV status. However, only a few studies have performed coimmunostaining to identify the M1 and M2 phenotypes [61]. M2 macrophages are involved in the enhancement of immunosuppression through the stimulation of Tregs and the secretion of TGF-b, TNF-a, and IL-10, leading to the creation of a favorable tumor microenvironment. Recently, Ou et al. indicated that CD68+ macrophage infiltration in HPV−SCC may consist mostly of M2 macrophages [61]. In HPV−HNSCC, the increased proportion of M2 cells could be one factor underlying the improvement in OS.

Because Foxp3 Tregs inhibit the activity of CD8+ T cells, these cells have been examined in many studies, and their relationship with the tumor microenvironment in HPV−induced SCC remains ambiguous. Indeed, in contrast with expectations, most studies reported that an increase in Treg infiltration, especially in HPV+ SCC, was associated with improved OS [15,24,34,35,36,39,53,54]. Irrespective of HPV status, some authors have reported similar findings in HNSCC [66,67]. In fact, the high infiltration of Foxp3 Tregs may inhibit the protumoral effects of inflammatory immune cells and may function as favorable prognostic markers at some tumor sites, whereas at other tumor sites, Treg infiltration may be linked to poor OS due to their conventional regulatory function [41,68,69]. The activation of Tregs may be associated with MDSC infiltration because DC subpopulations are known to stimulate Tregs in HNSCC [69]. However, we cannot advance any hypotheses about the potential role of DC in the development of HPV−induced SCC because of the low number of studies on this topic in the current literature. Future studies that aim to investigate the role of Foxp3 Tregs in HPV−induced HNSCC should examine other immune cell populations, such as MDSCs.

This systematic review permitted the identification of many factors that should be taken into consideration in the analysis of results, the comparison of studies and the future establishment of immune models.

First, many authors have combined patients with oropharyngeal and non-oropharyngeal SCC into a single group. However, HNSCC includes malignancies that arise from functionally and anatomically distinct tumor sites with different characteristics. The most blatant example concerns oropharyngeal histology, as the Waldeyer ring is composed of preexisting lymphoid tissue that is characterized by a higher sensitivity to the immune response [49]. In that respect, oropharyngeal SCC is usually heavily infiltrated by lymphocytes, in contrast to other types of HNSCC such as oral carcinoma [49,69]. Additionally, recent data have shown the heterogeneous molecular and immunological tumor profile of HNSCC at different anatomical locations [70]. Differences in the prognostic value of TIC might reflect these different biological factors, making it likely that TICs exhibit different properties depending on the tumor site and the histological and molecular subtype.

In addition to the impact of the anatomical location of tumors, the consumption of tobacco and alcohol may also have a critical impact on the significance of TICs. Indeed, because cohorts of HPV+ patients are usually small, most authors do not consider these factors to be important in the development of TICs. However, it has been suggested that tobacco and alcohol consumption are capable of stimulating the mucosal recruitment of LCs, impacting the local immune response during SCC development [18]. In the same vein, Geng et al. demonstrated that tobacco smoking and, in particular, nicotine, are known to impair the responsiveness of T cells to antigenic stimulation, while other authors found that smoking is associated with a lower number of CD8+ T cells in tissue [46,71,72].

Second, the analysis of TICs could ideally consider both the stromal and intraepithelial compartments because the level of infiltration could vary substantially between these two compartments, leading to differences in prognostic value. Some authors did not specify the compartment used for analysis, which makes an analysis of the results difficult [40,41,44].

A third important point regarding the results analysis is the method used to detect HPV infection. In clinical practice, HPV infection is detected through p16 immunostaining in many centers. Some authors have used this approach to compare data from HPV+ and HPV− SCC, while others have used PCR and other direct methods of DNA identification. However, it is well known that some tumors can be HPV+/p16− or HPV−/p16+, which can be related to the lack of specificity of p16 in identifying HPV infection. As demonstrated in two studies, the use of p16 immunostaining versus DNA detection may lead to differences in the results, biasing the conclusions of related studies [40,54].

This systematic review also showed that some immune cells are rarely studied, such as eosinophils or natural killer cells (NK cells). However, NK cells have been identified as immune cells that may directly kill both HPV+ and HPV− HNSCC tumor cells [72,73]. This anti-tumor activity of NK cells could be significantly enhanced by cetuximab or avelumab, particularly in cells with higher baseline EGFR or PD-L1 expression [73]. Clinically, irrespective of HPV status, the low number of tumor-infiltrating CD56+ NK cells is correlated with significantly decreased OS, distant metastasis-free survival and local progression-free survival [17].

Finally, the studies that investigated the impact of HPV infection on OS included patients subject to different treatment methods, including surgery, chemoradiation, radiotherapy and immunotherapy. These different therapeutic methods may significantly impact the OS of patients irrespective of HPV status. This point must be considered in analyses of OS in future large cohort studies.

In conclusion, current knowledge regarding the immune environment of HPV−induced HNSCC is not sufficient for the establishment of a clear pathophysiological model. Although some evidence exists in terms of the roles of CD4+ and CD8+ T cells and their related impact on OS or RFS, many uncertainties persist regarding the role and prognostic value of Tregs, macrophages, DCs and other uninvestigated cells. The poor quality and the low number of available studies, the small number of HPV+ patients included in these studies and the lack of consideration of cofactors that can impact the TIC composition may explain the current inability to establish an immunological model that can better predict the prognosis of HPV−induced oropharyngeal SCC. Future studies are needed to understand the complex interaction between tumors and their immune environment. These studies should carefully consider a rigorous methodological approach for HPV detection and should include a large number of patients with well-defined tumor locations. A large panel of immune cells and the use of specific coimmunostaining should be considered in future work in order to establish a precise immunological overview of HPV+ and HPV− SCC.

## Figures and Tables

**Table 1:** Immune cell roles.

Cells	Cell Marker	Theoretical Roles in Head & Neck Squamous Cell Carcinoma	Findings
CD8± T cells	CD8	1. Detection of neoplastic cells and cytotoxic effect through MHC class I binding.	Number of CD8+ T cells increases throughout tumor progression.
[9,13,18,23]		2. Recruitment of Treg lymphocytes.	The infiltration CD8+ T cells is usually associated with a better
		3. Upregulation of PD-L1 on tumor cells through an interferon (IFN)-γ-dependent	host response to the tumor.
		Manner.	Higher CD8+ T cell infiltration is associated with better OS.
CD4± T cells	CD4	The role of CD4+ helper T cells is unclear because a wide range of CD4+ cell subsets	The high infiltration of CD4+ T cells is associated with controversial results.
[13,18,23]		with different functions exists.	Some authors reported that in HPV−negative patient cohorts, a high level of CD4+
		1. Th-1 lymphocytes may activate cytotoxic lymphocytes.	TILs was associated with better OS and DFS, while contradictory
		2. Th-2 lymphocytes stimulate humoral immunity and activate eosinophils.	results were found for HPV+ patients.
		3. Th-17 lymphocytes may have a Th-1 phenotype in the tumor microenvironment.	The role of Th-17 in HNSCC is still controversial.
Regulatory	Foxp3	1. Maintenance of the immunological tolerance to host tissues (suppressors of the anti-tumor	Tregs may promote tumor progression.
T cells		immune response.)	The involvement of Tregs in HNSCC is controversial: some studies
[13,15,18,23]		2. Suppression of cytotoxic lymphocytes & other cells (MDSCs, etc.).	showed that an increased level of Tregs is linked with a worse prognosis.
			Others reported that high Treg counts are associated with a better prognosis.
			Immune escape is achieved by producing IL-10 and TGF-β and by consuming IL-2.
Dendritic cells	Immature: CD1a	1. Presentation of tumor-associated antigens to the immune system (MHC class I & II)	DC infiltration increases throughout tumor progression.
LC & MDSC	Immature: S-100	& stimulation of T cell differentiation.	The role of LC in HNSCC is controversial: some studies showed
[13,18,19,20,21,22,25]	Mature: CD83	2. Immunosuppressive agents such as IL-10 and TGF-β convert immature DCs into	that DC infiltration decreases in HNSCC, while others observed
	LC: CD207	tolerogenic DCs, inducing antigen-specific T cell tolerance through the activation	that it was higher in fibrotic oral submucosal areas of HNSCC.
		of Tregs, the silencing of differentiated antigen-specific T cell tolerance and the differentiation	
		of naïve CD4+ T cells into Tregs.	
Macrophages	CD68	Presentation of tumor-associated antigens to immune system and the regulation of inflammation.	Macrophage infiltration increases throughout tumor progression.
[13,14,18,23]	M2: CD68/CD163	M1: Activation of cytotoxic CD8+ T cells and Naïve CD4+ T cell differentiation into	Invasion, intratumoral microvessel density & angiogenic factors
		Th1 effector cells (antitumor effects).	(VEGF) were positively associated with the level of macrophage infiltration.
		M2: Stimulation of Treg differentiation; the secretion of some factors (TGF-b, TNF-a,	Macrophages facilitate tumor matrix generation & angiogenesis via
		IL-10) creates a favorable environment for tumor growth and immunosuppression	elimination of extravascular fibrin deposits. They also stimulate HIF-1α
		promotion.	expression, making the tumor more invasive and aggressive.
Natural killers	CD56	1. Cytotoxic T lymphocyte activation	NK cells are a cell therapy product capable of mediating direct and
[17,18]		2. Direct anti-tumoral activity	antibody-dependent cell-mediated cytotoxicity. They may directly kill
			HPV− & HPV+ HNSCCs. The low numbers of tumor-infiltrating CD56+ NK cells
			is correlated with significantly decreased OS, distant metastases-free
			survival and local progression-free survival (Lu).

Abbreviations: DFS = disease free survival; IFN = interferon; IL = interleukin; HIF = hypoxia inducible factor; HNSCCs = head neck squamous cell carcinomas; LC = Langerhans cell; M1-2 = macrophage type 1-2; MDSCs = myeloid-derived suppressor cells; MHC = major histocompatibility complex; OS = overall survival; PD-L1 = Programmed death ligand 1; TGF = tumor growth factor; Th = T helper; TILs = tumor infiltrated lymphocytes; Treg = T regulatory cells; VEGF = vascular endothelial growth factor. This table was created based on several references.

**Table 2:** Peripheral blood immune cell concentration of patients with head and neck squamous cell carcinoma according to HPV status.

Authors	Specimens	Blood Cells & Antigens	Antigen Blood Expression	Findings
Heusinkveld	Oropharynx, Oral,	Peripheral isolated blood monocytes		1. HPV16-specific T cell response comprised a broad repertoire of CD41 T-helper
2012 [27]	Hypopharynx	T cells specific to HPV oncoproteins	N = 17/47	cells (type 1 and type 2), CD41 Tregs and CD81 T cells reactive to HPV16.
	Carcinoma: 50	IFN-Υ expression	N = 5/17	2. Stimulated CD4+ & CD8+ T cells in patients with HPV+ HNSCCs produce
	*HPV+: 12-HPV−: 29*	T cells specific to p53 protein	N = 7/45	IFN-Υ, IFN-a, IL-4, IL-5.
		IFN-Υ expression	N = 1/7	
		Blood CD25þFoxp3þ cells	HPV+ = HPV−	
		Cytokine production of isolated CD4+		
		& CD8+ T cells of HPV+ tumors	IFN-Υ, IFN-a, IL-4, IL-5	
Al-Taei,	Oropharynx & NT	Treg CD4+CD25hiFoxp3+	C > NT; pre > posttreatment	1. Immunosuppression may contribute to the reduction in the HPV−specific T cell
2013 [28]	Carcinoma: 20	MDSC CD14−Hla- Dr−CD15hi	C > NT; pre > posttreatment	response posttreatment (i.e., surgery, chemoradiation, immunotherapy), indicating that
	*HPV+/p16+: 9*	Memory CD3+ T Cells	Pre > posttreatment	HPV−targeted immunotherapy in oropharyngeal SCC patients posttreatment could require
	*HPV−/p16−: 2*	Naïve T Cells, CD3+ T cells	Pre > posttreatment	multiple strategies to boost T cell immunity and to overcome the influence of
	*HPV−/p16+: 1*			immunosuppressive cells.
Parikh,	Oropharynx	Circulating cells before CRT		1. CRT decreased circulating T cells and markedly elevated MDSCs.
2014 [29]	Carcinoma: 22	HPV±: CD4+ T Cells; Treg	C = N	2. PD-1 expression on CD4+ T cells increased after CRT as well as CD45RO+.
	*HPV+: 20-HPV−: 2*	HPV±: CD8+ T Cells	N > C	3. CRT suppressed circulating immune responses in patients with HPV+ tumors by
		HPV±: MDSC	C > N	unfavorably altering effector cells: suppression of immunocyte ratios and upregulation of PD-1
		Circulating cells after CRT		expression on CD4+ T cells were noted.
		HPV±: CD4+; CD8+ T Cells; & Treg	Pre > post	
		HPV±: MDSC	Post > pre	
		PD-1 expression after CRT		
		PD-1 expression of CD4+ T Cells	Post > pre	
		CD45RO+ CD4+ T cells	Post > pre	
		PD-1 expression of Treg	Pre = post	
		PD-L2 expression of AP cells	Post > pre	
Lukesova	Oropharynx & Oral	CD3-CD56+CD16+ NK	HPV+ > HPV−	1. Patients with HPV+ oral & oropharyngeal SCC have higher blood concentrations of
2014 [30]	Carcinoma: 60	CD4+ & CD8+ T cells	HPV+ = HPV−	CD3-CD56+CD16+ NK. 2. A higher Treg level & a lower CD8/Treg ratio influenced
	*HPV+/p16+: 27*	CD19+ cells & Tregs	HPV+ = HPV−	OS independently of HPV status and age. 3. Patients with HPV+ tumors had better OS
	*HPV+/p16−: 3*	CD4+/CD8+ ratio	HPV+ = HPV−	and RFS than those with HPV− tumors. Among HPV+ patients, a high Treg blood
	*HPV−: 30*	Tregs elevated serum level	37/60	concentration was associated with better OS and RFS.
Masterson	Oropharynx	Pretreatment CD4+ against E2	N: 8/30	1. CD8+ T cells enhanced immunoreactivity to antigen E7 was linked to improved OS.
2016 [31]	Carcinoma: 51	Pretreatment CD8+ against E6	N: 18/30	2. An increase in Treg level after treatment suggests that immunosuppression can
	*HPV+: 41-HPV−: 10*	Pretreatment CD8+ against E7	N: 21/30	contribute to a reduced HPV−specific cell-mediated response.
	*Normal: 11*	Pretreatment CD4+CD25+ Tregs	HPV+ = HPV−	
		Posttreatment CD4+CD25+ Tregs	HPV+: post > pretreatment	
Ma,	Oropharynx & Oral	HPV±: MDSC CD11b-CD33	C > D > N	1. HPV infection increases the level of blood MDSCs, promoting the recruitment of
2017 [32]	Normal/dysplasia: 30/30			MDSCs into HNSCC areas.
	Carcinoma: 196			2. The blood level of MDSCs is higher for cT3/T4 tumors (stages II and III)
	HPV+: 47			than for cT1-T2 tumors (stage I).
Lechner	Oropharynx, Oral,	CD45+CD3+ lymphocytes	TB > NM; TB = NB	1. The tumor microenvironment of HNSCC is characterized by strong Treg infiltration
2017 [33]	Larynx, Hypopharynx,	CD45+CD4+ lymphocytes	T > TB; NM > TB; TB = NB	and elevated checkpoint molecule expression in T cell subsets.
	Sinus	CD8+ T cells	TB = T = NM = NB	2. These different cells are found in the circulation.
	Carcinoma: 34	Naïve T cells (CD45RA+/CCR7+)	TB > T	
	*HPV−: 26-HPV+: 8*	Effector memory T cells	T > TB; NM > TB; TB = NB	
	Normal: 7	Treg CD4+/CD25+/CD127low	TB > NB; T > TB; T > NM; TB = NM	
		Treg CD4+/CD39+	TB = NB; T > TB; TB = NM	
		Effector memory T cells CD45RA-/CCR7-	HPV+ = HPV−	
		Treg CD4+/CD25+/CD127low	HPV+ = HPV−	
		Checkpoint protein expression on T cells		
		PD-1 T Cells	T > TB; TB > NB; TB > NM	
		CTLA-4+	TB > NB	
		Effector memory T cells CD45RA-/CCR7-	HPV+ = HPV−	
		Treg CD4+/CD39+	HPV+ = HPV−	
		Treg CD4+/CD25+/CD127low	HPV+ = HPV−	

Abbreviations: C = carcinoma; CRT = chemoradiotherapy; CTLA = cytotoxic T-lymphocyte antigen; D = dysplasia; HNSCC = head neck squamous cell carcinoma; HPV = human papilloma virus; IFN = interferon; IL = interleukin; MDSC = myeloid-derived suppressor cell; N = number; NB = node blood; NK = natural killer; NM = normal mucosa; NT = normal tissue; OS = overall survival; PD-1 = programmed death-1; PD-L2 = programmed death ligand 2; RFS = recurrence-free survival; T = tumor (TNS status); TB = tumor blood; Treg = regulatory T cells.

**Table 3:** CD8+ and CD4+ infiltration in HPV+ and HPV− Head & Neck Squamous Cell Carcinoma.

Authors	No. of Specimens	Tumor-Infiltrating Cells	Antigens/HPV	Findings	
Wansom	Oropharynx	CD8+ T cells	HPV+ = HPV−	1. Degree of CD8+ T cell infiltration did not differ by HPV status in oropharyngeal SCC.	
2011 [34]	Carcinoma: 38			2. High infiltration of CD8+ T cells was associated with better OS.	
	*HPV+:25-HPV−:13*				
Badoual	Oropharynx, Oral,	Intratumoral CD8+ T cell infiltration	HPV+ > HPV−	1. HPV+ HNSCC tumors had a higher number of infiltrating CD8+ T cells than did HPV− tumors.	
2012 [35]	Larynx, Hypopharynx	Antigen expression (CD8+, CD4+ & Foxp3+)		2. PD-1+ T cells was positively correlated with a favorable clinical outcome.	
	Carcinoma: 64	HLA-DR, CD38 & Tim-3	PD1+ > PD1- T cells; HPV+ = HPV−		
	*HPV+: 32-HPV−: 32*				
Nasman	Oropharynx	CD8+ tumor infiltration	Gr1,3 > Gr2,4; HPV+ > HPV−	1. HPV+ oropharyngeal SCC had a higher number of infiltrating CD8+ T cells than did HPV− SCC.	
2012 [36]	Carcinoma: 83			2. A high CD8+Foxp3+/tumor-infiltrating lymphocyte ratio was correlated with a	
	*Gr1: HPV+, GP: 31*			better 3-year RFS.	
	*Gr2: HPV+, PP: 21*			3. A high infiltration of CD8+ in HPV+ SCC was associated with a better	
	*Gr3: HPV−, GP: 11*			RFS than a low infiltration of CD8+ in HPV− SCC.	
	*Gr4: HPV−, PP: 20*				
Jung	Oropharynx	CD8+ T cells (stroma)	HPV+ > HPV−	1. Stroma of HPV−positive tumors was frequently and strongly infiltrated by CD8+ and	
2012 [37]	Carcinoma: 17	CD4+ T cells (stroma)		CD3+ T cells. 2. CD8+ infiltration improved OS and RFS. 3. CD3+ infiltration improved OS.	
	*HPV+: 10-HPV−:7*			4. CD4+ staining is more important in the stroma of HPV+ SCCs.	
Rittà	Oropharynx	CD3+; CD4+; CD8+ (intratumoral)	HPV+ > HPV−	1. HPV16+ oropharyngeal SCC has a better prognosis than HPV− SCC.	
2013 [38]	Carcinoma: 22			2. No difference was found in the extent of tumor infiltration by CD25+, FoxP3+, CD3+, C68+,	
	*HPV+: 10-HPV−: 12*			CD20+, CD8+, or CD4+ cells according to HPV status. 3. HPV16 load values equal or greater	
				than 10-1 copies per cell were associated with higher densities of TIL CD3+ and Foxp3+ cells.	
Russell	Oropharynx, Oral, Sinus,	Intratumoral CD8+ T cells	HPV+ > HPV−	1. HPV+ HNSCC exhibits a significantly increased infiltration of intratumoral CD8+ T cells	
2013 [39]	Larynx, Hypopharynx	Stromal CD8+ T cells	HPV+ = HPV−	compared with HPV− HNSCC. 2. qRT-PCR data demonstrated a general pattern of increased	
	Carcinoma: 34	Expression of genes/antigens (qRT-PCR)	C > N (IL-2: HPV+ > HPV−)	immune activation and suppression mechanisms in HPV+ samples.	
	*HPV+: 9-HPV−: 26*	IL-2, 4, 8, 12	Associated with upregulation		
	Normal: 7		of PDL1, B7H4, & FasL		
Nordfors	Oropharynx	CD8+ T cells	HPV+ > HPV−	1. In HPV+ and HPV+p16a+ SCC, a higher number of CD8+ TILs was correlated with better OS and RFS.	
2013 [40]	Carcinoma: 280	CD4+ T cells		2. In HPV− SCC, a higher number of CD8+ TILs was correlated with better OS.	
	*HPV+: 220-HPV−: 60*			3. The number of CD4+ TILs was not correlated with better OS.	
Ward	Oropharynx	CD8+ T cells	HPV+(TILhigh) > HPV+(TILlow)	1. HPV+ OPSCC exhibits higher levels of TILs than HPV− OPSCC.	
2014 [41]	Carcinoma:270	CD4+T cells	HPV+(TILlow) = HPV−	2. HPV+ OPSCC with a high level of TILs	
	*HPV+:149-HPV−:121*			has a better prognosis than HPV+ OPSCC with a low level of TILs.	
				3. HPV+ OPSCC with a low level of TILs has the same	
				prognosis as HPV− OPSCC.	
Balermpas	Oropharynx, Oral,	CD8+ T cells (intratumoral, stromal)	HPV+ = HPV−	1. High CD3+ and CD8+ infiltration was associated with better OS, PFS, and distant metastasis	
2014 [42]	Larynx, Hypopharynx	CD4+ T cells (intratumoral, stromal)		-free survival. Infiltration of CD4+, CD8+ and CD3+ was similar in HPV+ & HPV− SCC.	
	Carcinoma:101	CD3+ (intratumoral, stromal)		2. CD4+ expression was not associated with a good prognosis.	
Krupar		Oropharynx	CD3+ (intra and peritumoral)	HPV+ > HPV−	1. In HPV+ tumors, we found significantly increased peritumoral infiltration of CD3+ T cells
2014 [43]		Carcinoma: 33	CD8+ T cells (intra and peritumoral)		and trends toward higher peritumoral CD4+ and CD8+ T cell infiltration.
		*HPV+: 16-HPV−: 17*	CD4+ T cells (intra and peritumoral)		2. No differences between the two tumor types were observed in terms of Th17+ TIsL.
			IL-17 (TH17 cells) (intra and peritumoral)		3. The CD8/CD4 ratio was significantly higher in the intratumoral compartment of HPV+ tumors.
					4. A significantly lower percentage of Th17+ T cells in the intratumoral compartment was noted in
					HPV+ cases.
Partlova		Oropharynx, Oral,	CD8+ T Cells; CD8+IFN-g+;	HPV+ > HPV−	1. HPV+ HNSCC showed significantly higher numbers of infiltrating IFN-g+ CD8+ &
2015 [44]		Larynx, Hypopharynx,	% of CD8+ IFN-g+ among other T Cells	HPV+ > HPV−	IL-17+ CD8+ T cells.
		Submaxillary gland	CD8+IL-17+ T Cells	HPV+ > HPV−	2. The infiltration of immune cells was associated with an increased secretion of
		Carcinoma: 44	% of CD8+IL-17+ T Cells among other T Cells	HPV+ > HPV−	proinflammatory cytokines.
		*HPV+: 20-HPV−: 24*	In vitro cytokine production from unstimulated tumor-		3. The high level of CXCL12 was associated with a higher node status.
			derived cell culture supernatants		
			CCL-17; 21	HPV+ > HPV−	
			CXCL9; 10; 12; IL-1b, 2, 17, 23; IFN-g	HPV+ = HPV−	
			In vitro cytokine production from stimulated tumor-		
			derived cell culture supernatants		
			IL-10, 17, 21; TNF-a; IFN-g	HPV+ > HPV−	
			mRNA expression in tumor samples		
			Cox-2	C > LN; C > NN; HPV− > HPV+	
			PD-1	C = LN = NN; HPV+ > HPV−	
			PD-L1	C > NN; HPV+ = HPV−	
			Tim-3	C > LN > NN; HPV+ = HPV−	
Oguejiofor		Oropharynx	Stromal & tumoral CD3+CD8+ T cells	HPV+ > HPV−	1. HPV+ OPSCC has higher infiltration of CD3+CD8+ T cells in tumor
2015 [45]		Carcinoma: 218			& stromal areas than HPV− OPSCC.
		*HPV+: 139-HPV−: 79*			2. CD3+CD8+ stromal infiltration was associated with increased OS.
Balermpas		Oropharynx, Oral,	Intratumoral & stromal CD8+ T cell infiltration	Oropharynx > hypopharynx & oral	1. HPV+ HNSCC has higher infiltration of CD8 T cells than HPV− HNSCC.
2016 [46]		Hypopharynx		HPV+ > HPV−	2. OPSCC has higher CD8+ infiltration than non-oropharyngeal SCC.
		Carcinoma: 161		cT1-2 > cT3-4	3. High CD8+ infiltration was associated with better OS & RFS.
		*HPV+: 100 - HPV−: 61*			4. Low CD8+ infiltration was associated with a high risk of metastases.
Van Kempen		Oropharynx	Intratumoral CD8+	HPV+ > HPV−	1. HPV+ OPSCC have higher infiltration of CD8+ T cells than HPV− OPSCC.
2016 [47]		Carcinoma: 262			
		*HPV+: 44-HPV−: 218*			
Kim	Oropharynx, Oral,	CD8+ tumor oropharyngeal infiltration	HPV+ > HPV -	1. CD8 T cell infiltration was higher in HPV+ OPSCC than in HPV− OPSCC.	
2016 [48]	Larynx, Hypopharynx	Checkpoint protein expression		2. High CD8+ infiltration was associated with better OS & RFS in HPV+ OPSCC.	
	Carcinoma: 402	PD-1+ TILs & intratumoral LAG-3+ TILs	HPV+ > HPV -		
	*HPV+: 198-HPV−: 204*				
Nguyen	Oropharynx, Oral,	CD8+ T cells (intratumoral)	HPV+ > HPV−	1. Higher CD4 and CD8 TIL levels were associated with improved OS & RFS.	
2016 [49]	Larynx, Hypopharynx	CD4+ T cells (intratumoral)		2. CD4 & CD8 infiltration was higher in HPV+ than in HPV− HNSCC.	
	Carcinoma: 278			3. Higher numbers of CD4 & CD8 T cells were observed in OPSCC than in HNSCC.	
	*HPV+:89-HPV−:218*			4. Lower numbers of CD4 & CD8 T cells were observed in HPV− OPSCC than in HPV− OPSCC.	
Poropatich	Oropharynx, Oral	Intratumoral CD8+ T cells	HPV+p16+ > HPV−p16−	1. HPV+ oral & OPSCC tumors have higher infiltration of CD8 T cells than HPV− tumors.	
2017 [50]	Carcinoma: 40			2. High CD8+ infiltration was associated with better OS & RFS in HPV+ oral & OPSCC tumors. *	
	*HPV+: 21-HPV−: 19*				
Sivars	Oropharynx	Intratumoral CD8+ T cells	HPV+DNA = HPV−DNA	1. CD8+ infiltration is higher in HPV+ tumors than in HPV− tumors.	
2017 [51]	Carcinoma: 69		p16+ > p16−	2. High CD8+ infiltration is associated with better OS in HPV+ oropharyngeal SCC.	
	*HPV+: 32-HPV−: 37*		HPV+p16+ > HPV−DNA		
Kansy	Not specified	Infiltration of CD8A+	HPV+ > HPV−	1. CD8A+ & CD8B+ T cell infiltration is higher in HPV+ HNSCC than in HPV− HNSCC.	
2017 [52]	Carcinoma: 40	Infiltration of CD8B+	HPV+ > HPV−	2. PD-1 expression is higher in CD8+ T cells of HPV+ HNSCC than in those of HPV− HNSCC.	
	*HPV+: 20-HPV−: 20*	PD-1 expression (qRT-PCR)	C > N; HPV+ > HPV−		
	Normal: 4				

Abbreviations: CCL-25 = Chemokine (C-C motif) ligand 25; C/N = carcinoma/normal tissue; CXCL = Chemokine (C-X-C motif) ligand; FasL = Fas ligand; GP - PP = good - poor prognostic; HLA-DR = Human Leukocyte Antigen – DR isotype; HPV = human papilloma virus; IFN = interferon; LAG-3 = Lymphocyte-activation gene 3 protein; LN/NM = lymph node/normal node; OPSCC = oropharyngeal SCC; OS = overall survival; IL = interleukin; PD-1 = programmed cell death 1; PD-L1 = Programmed death ligand 1; PFS = progression-free survival; qRT-PCR = Real-Time Quantitative Reverse Transcription PCR; RFS = recurrence-free survival; (HN)SCC = (Head & Neck) squamous cell carcinoma; TILs = tumor-infiltrating lymphocytes; Tim-3 = T cell immunoglobulin and mucin-domain containing-3; Th = T helper; TNF-a = Tumor Necrosis Factor alfa. *However, the expression of IL2, 4, 8, 12 was not associated with specific immune cells These findings may or may not be associated with CD8+ T cell infiltration. * mRNA expression and cytokine production were not associated with specific immune cells. In other words, these findings may or may not be associated with CD8+ T cell infiltration. ** Statistical analyses were performed on oral & oropharyngeal tumor samples without distinction.

**Table 4:** Foxp3 T regulatory cell infiltration in HPV+ and HPV− head & neck squamous cell carcinoma.

Authors	No. of Specimens	Tumor-Infiltrating Cells	Antigens/HPV	Findings
Partlova	Oropharynx, Oral,	Treg	HPV− > HPV+	1. HPV+ HNSCC showed a slightly lower proportion of Tregs than HPV− HNSCC.
2015 [12]	Larynx, Hypopharynx,	In vitro cytokine production from tumor		2. The infiltration of immune cells (i.e., Treg) is associated with
	Submaxillary gland	unstimulated derived cell culture supernatants		increased proinflammatory cytokine secretion.
	Carcinoma: 44	CCL-17; 21	HPV+ > HPV−	3. The high level of CXCL-12 was associated with a higher node status *
	*HPV+: 20-HPV−: 24*	CXCL9; 10; 12; IL-1b, 2, 17, 23; IFN-g	HPV+ = HPV−	
		In vitro cytokine production from tumor		
		stimulated derived cell culture supernatants		
		IL-10, 17, 21; TNF-a; IFN-g	HPV+ > HPV−	
		mRNA expression level in tumor samples		
		Cox-2	C > LN; C > NN; HPV− > HPV+	
		PD-1	C = LN = NN; HPV+ > HPV−	
		PD-L1	C > NN; HPV+ = HPV−	
		Tim-3	C > LN > NN; HPV+ = HPV−	
Punt	Oropharynx	CD3+Foxp3+ Treg	HPV+ > HPV−	1. Foxp3 Treg infiltration is higher in HPV+ OPSCC than in HPV− OPSCC.
2016 [54]	Carcinoma: 162			2. A high number of infiltrated T cells was associated with improved DFS
	*HPV−: 99-HPV+: 63*			in the HPV+ OPSCC group.
Nguyen	Oropharynx, Oral,	Foxp3+ T cells (intratumoral)	HPV+ > HPV−	1. The Foxp3 intratumoral infiltration is higher in HPV+ than in HPV− HNSCC.
2016 [49]	Larynx, Hypopharynx			2. Foxp3 infiltration is higher in OPSCC than in other SCC types.
	Carcinoma: 278			
	*HPV+:89-HPV−:218*			
Kindt	Oropharynx, Oral,	Stromal infiltration of Foxp3+ Tregs	C > SD > LD > N	1. Foxp3+ T cell infiltration increased with tumor progression, with the increase being more
2017 [24]	Larynx, Hypopharynx		HPV+/p16+ =	important in HPV+ patients. 2. A high Foxp3+ T cell number in the stromal compartment
	Carcinoma: 110		HPV+/p16− & HPV−	is associated with longer recurrence-free survival and overall survival.
	*HPV negative: 82*	Intraepithelial infiltration of Foxp3+ Tregs	C>N; SD, LD > N	3. Foxp3+ T cell number improved the prognostic value of tumor stage.
	*HPV+/p16−: 12*		-HPV+/p16+ >	4. Stromal Foxp3+ T cell number is a strong prognostic factor independent of
	*HPV+/p16+: 6*		HPV+/p16− & HPV−	other risk factors i.e., tobacco, alcohol, HPV status.
	N/LD/SD: 10/43/45			
Lechner	Oropharynx, Oral, Sinus,	CD4+/CD39+ Tregs	T = NM;T>TB; TB = NM	1. HNSCC is characterized by a strong infiltration of Tregs irrespective to the HPV status.
2017 [33]	Larynx, Hypopharynx,	CD4+/CD25+/CD127low Tregs	HPV+ = HPV−	2. HNSCC is characterized by high checkpoint molecule expression in T cell subsets.
	Carcinoma: 34	Checkpoint protein expression on T cells		These different cells are found in the circulation.
	*HPV−: 26-HPV+: 8*	PD-1 T cells	T>TB; TB>NM	
	Normal: 7	CTLA-4+	T = NM	
		CD4+/CD39+ Tregs	HPV+ = HPV -	
		CD4+/CD25+/CD127low Tregs	HPV+ = HPV−	
Seminerio	Oropharynx, Oral,	Foxp3+ Tregs	HPV+ = HPV -	1. No difference was found between HPV+ & HPV− SCC in terms of the infiltration of Foxp3+ Tregs.
2019 [15]	Larynx, Hypopharynx			2. A high infiltration of Foxp3+ Tregs in intratumoral & stromal compartments was
	Carcinoma: 205			associated with longer RFS & OS. 3. FoxP3+ Treg stromal infiltration, tumor
	HPV+: 35-HPV−: 127			stage and histological grade independently influenced patient prognosis.

Abbreviations: CCL-25 = Chemokine (C-C motif) ligand 25; C/N/LD/SD = carcinoma/normal tissue/low dysplasia/severe dysplasia; CXCL = Chemokine (C-X-C motif) ligand; FasL = Fas ligand; GP - PP = good - poor prognostic; HLA-DR = Human Leukocyte Antigen – DR isotype; HPV = human papilloma virus; IFN = interferon; LAG-3 = Lymphocyte-activation gene 3 protein; LN/NM = lymph node/normal node; OPSCC = oropharyngeal SCC; OS = overall survival; IL = interleukin; PD-1 = programmed cell death 1; PD-L1 = Programmed death ligand 1; PFS = progression-free survival; qRT-PCR = Real-Time Quantitative Reverse Transcription PCR; RFS = recurrence-free survival; (HN)SCC = (Head & Neck) squamous cell carcinoma; TIL = tumor-infiltrating lymphocytes; Tim-3 = T cell immunoglobulin and mucin-domain containing-3; Th = T helper; TNF-a = Tumor Necrosis Factor alfa. * mRNA expression and cytokine production were not associated with specific immune cells. In other words, these results may or may not be associated with Treg infiltration.

**Table 5:** Macrophage infiltration in HNSCC according to HPV status.

Authors	No. of Specimens	Tumor-Infiltrating Cells	Antigens/HPV	Findings
Ritta	Oropharynx, Oral,	CD68+ macrophages	HPV+ = HPV−	1. HPV+ HNSCCs do not have higher CD68+ macrophage infiltration than HPV− HNSCC.
2009 [55]	Larynx			2. A direct correlation between macrophage infiltration and tumor proliferation index
	Carcinoma: 59			was observed irrespective of the tumor subset.
	*HPV+: 27-HPV−: 32*			
Wansom	Oropharynx	CD68+ macrophages	HPV+ = HPV−	Degree of CD68+ macrophage infiltration does not differ by HPV status in OPSCC.
2011 [34]	Carcinoma: 38			
	*HPV+:25-HPV−:13*			
Lyfor-Pike	Oropharynx	CD68+PD1+	C > N; HPV+ > HPV−	The tumoral infiltration of CD68+ macrophages is higher in HPV+ OPSCC
2013 [56]	Carcinoma: 27			than in HPV− OPSCC. CD68+ macrophages express high levels of PD-1.
	*HPV+: 20-HPV−:7*			
Russell	Oropharynx, Oral, Sinus,	Intratumoral CD68+ macrophages	HPV+ = HPV−	HPV+ HNSCC are not characterized by higher CD68+ macrophage infiltration
2013 [39]	Larynx, Hypopharynx,	Stromal CD68+ macrophages	HPV+ = HPV−	than HPV− HNSCC samples.
	Carcinoma: 34			
	*HPV+: 9-HPV−: 26*			
Partlova	Oropharynx, Oral,	Monocytes & macrophages	HPV+ = HPV−	HPV+ tumor samples are not characterized by higher monocyte &
2015 [44]	Larynx, Hypopharynx,			macrophage infiltration than HPV− tumor samples.
	Submaxillary gland			
	Carcinoma: 44			
	*HPV+: 20-HPV−: 24*			
Yu	Oral	Stromal macrophages CD68+/CD163+	C > N; HPV+ = HPV−	The CD68+/CD163+ macrophage population increased
2015 [57]	Carcinoma: 86			in both HPV+ and HPV− HNSCC.
	*HPV+: 12-HPV−: 74*			
	Normal/dysplasia: 32/12			
Lee	Oropharynx	High OS & DFS	High CD68+ >	1. A high infiltration of CD68+ macrophages is associated with low OS & DFS.
2015 [58]	Carcinoma: 53		low CD68+ macrophages	2. The number of CD68+ macrophages could be determining factors for CRT
	*HPV+: 39-HPV−: 14*			outcomes in patients with HPV+ OPSCC.
Nguyen	Oropharynx, Oral,	CD68+ (intratumoral)	HPV+ > HPV−	1. HPV+ HNSCC has a higher CD68+ macrophage infiltration than HPV− HNSCCs.
2016 [49]	Larynx, Hypopharynx			2. CD68+ macrophage infiltration was higher in OPSCCs than in other SCCs.
	Carcinoma: 278			
	*HPV+:89-HPV−:218*			
Seminerio	Oropharynx, Oral,	Stromal CD68+ macrophages	C > SD > LD > N	1. CD68+ macrophage numbers increased during HNSCC progression in intraepithelial
2017 [14]	Larynx, Hypopharynx		HPV+/p16+ =	& stromal compartments. 2. A higher density of CD68+ macrophages was observed
	Carcinoma: 110		HPV+/p16− & HPV−	in advanced stages. 3. Patients with HPV+/p16+ HNSCC had a higher CD68+ macrophage
	*HPV−: 82*	Intratumoral CD68+ macrophages	C > SD > LD >N	density than those with HPV− HNSCC. 4. High CD68+ macrophage numbers in
	*HPV+/p16−: 12*		HPV+/p16+ >	the intratumoral compartment were associated with shorter patient OS.
	*HPV+/p16+: 6*		HPV+/p16− & HPV−	5. CD68+ macrophage infiltration was a strong & independent prognostic factor of HNSCC.
	N/LD/SD: 10/43/45			
Oguejiofor	Oropharynx	Intratumoral CD68+	HPV+ > HPV−	1. HPV+ OPSCC have higher CD68+ macrophage infiltration in intratumoral but not in
2017 [59]	Carcinoma: 124	Stromal CD68+	HPV+ = HPV−	stromal compartments than HPV− OPSCC. 2. High stromal infiltration of CD68+PD-L1+
	*HPV+: 75-HPV−: 49*	CD68+ PD-L1+ stromal density	HPV− > HPV+	is associated with better OS in patients with HPV− tumors.
Welters	Oropharynx	DC-like macrophages	HPV+ > HPV−	1. DC-like macrophage infiltration was higher in HPV+ OPSCC,
2017 [60]	Carcinoma: 97			which was correlated with better OS and a low risk of lymph node metastases.
	*HPV+: 57-HPV−: 40*			
Ou	Oropharynx & non-	Intratumoral macrophage density	Oropharynx > non-oropharynx	1. HPV− HNSCC has a higher CD68+ macrophage intratumoral density than HPV− tumors.
2018 [61]	oropharynx		N2-N3 > N0, N1	2. There is a trend of a higher proportion of the M2 population in the CD68+ macrophage
	Carcinoma: 95		HPV− > HPV+	infiltration of HPV− tumors. 3. Low M2 macrophage density was associated
	*HPV+: 27-HPV−: 68*			with an improved 5-year disease free survival in patients treated by CRT.
Schneider	Oropharynx, Oral,	Oropharynx CD163+ macrophages	p16+ = p16−	1. CD168+ macrophage infiltration did not differ between p16+ and p16− HNSCC.
2018 [62]	Larynx, Hypopharynx	CD163+ macrophages	Oropharynx = hypopharynx =	
	Carcinoma: 136		Larynx = oral: p16+ = p16−	
	*p16+: 24-p16−: 112*			

Abbreviations: C/N/LD/SD = carcinoma/normal tissue/low dysplasia/severe dysplasia; CRT = chemoradiotherapy; HPV = human papilloma virus; M1/M2 = macrophages 1/2; OPSCC = oropharyngeal SCC; OS = overall survival; PD-1 = programmed cell death 1; PD-L1 = Programmed death ligand 1; PFS = progression-free survival; RFS = recurrence-free survival; (HN)SCC = (Head & Neck) squamous cell carcinoma; TIL = tumor-infiltrating lymphocytes.

**Table 6:** Infiltration of Langerhans cells and myeloid-derived suppressor cells in HNSCC according to HPV status.

Authors	No. of Specimens	Tumor-Infiltrating Cells	Antigens	Findings
Pereira	Oral	LC (Anti-S-100 antibody)		There was no association between the immunohistochemical labeling
2011 [63]	Carcinoma: 27	Stromal compartment infiltration	HPV− = HPV+	for LCs (S-100+) and HPV infection in oral SSC.
	*HPV−: 18-HPV+: 9*			HPV infection in oral SCC did not alter the infiltration of LCs.
Russell	Oropharynx, Oral, Sinus,	MDSC: CD11b, Arg-1, INOS, STAT3 antigen	C > N (HPV+ = HPV−)	1. Irrespective of HPV status, a higher expression of
2013 [39]	Larynx, Hypopharynx,	expression		MDSC antigens, including CD11b, Arg-1, INOS and STAT3, were found in HNSCC.
	Carcinoma: 34			
	*HPV+: 9-HPV−: 26*			
	Normal: 7			
Yu	Oral	Stromal MDSC CD11b/CD33	HPV+ = HPV−	1. Irrespective of HPV status, CD11b/CD33 MDSC populations
2015 [57]	Carcinoma: 86			increased in oral SCC.
	*HPV+: 12-HPV−: 74*			2. There was a positive correlation between the infiltration of MDSC &
	Normal/dysplasia: 32/12			PD-L1 expression
Partlova	Oropharynx, Oral,	MDSC	HPV+ > HPV−	1. HPV+ HNSCCs have a higher MDSC infiltration than HPV− HNSCCs.
2015 [44]	Larynx, Hypopharynx,	In vitro cytokine production from unstimulated tumor-		2. The infiltration of immune cells (i.e., MDSC) is associated with
	Submaxillary gland	derived cell culture supernatants		increased secretion of proinflammatory cytokines.
	Carcinoma: 44	CCL-17; 21	HPV+ > HPV−	3. HPV+ tumors had significantly lower expression of Cox-2 mRNA and
	*HPV+: 20-HPV−: 24*	CXCL9; 10; 12; IL-1b, 2, 17, 23; IFN-g	HPV+ = HPV−	higher expression of PD1 mRNA than did HPV− tumors.
		In vitro cytokine production from stimulated tumor-		4. The high level of CXCL-12 was associated with a higher node status.
		derived cell culture supernatants		* mRNA expression and cytokine production were not associated with
		IL-10, 17, 21; TNF-a; IFN-g	HPV+ > HPV−	*specific immune cells. In other words, these results may or may not be associated with MDSC*
		mRNA expression level in tumor samples		*infiltration.*
		Cox-2	C > LN; C > NN; HPV− > HPV+	
		PD-1	C = LN = NN; HPV+ > HPV−	
		PD-L1	C > NN; HPV+ = HPV−	
		Tim-3	C > LN > NN; HPV+ = HPV−	
Kindt	Oropharynx, Oral	CD1a+ LC & MIP-3α secretion		1. LC infiltration is increased in HNSCC but decreased with HPV infection.
2016 [64]	Larynx, Hypopharynx	Stromal compartment infiltration	C > SD > LD > N	2. LC number is an independent prognostic factor for HNSCC.
	Carcinoma: 125		HPV+/p16+ =	3. Intratumoral LC infiltration is positively associated with cT in oral SCC.
	*HPV−: 82*		HPV+/p16− & HPV−	4. LC infiltration is positively associated with node status.
	*HPV+/p16−: 21*	Intraepithelial compartment	C > N; SD, LD > N	5. Stromal infiltration of LCs is associated with better OS in HPV−
	*HPV+/p16+: 11*		HPV+/p16− > HPV+/p16+	but not HPV+ HNSCCs. 6. Intratumoral & stromal LC infiltration was
	N/LD/HD: 25/64/54			positively associated with better RFS in HPV− tumors.
Ma	Oropharynx & Oral	MDSC CD11b-CD33	C & D > N; HPV+: C > D > N	1. MDSC infiltration is higher in HPV+ oral & OPSCC samples than in dysplastic
2017 [32]	Carcinoma: 376	IncRNA expression (MDSC recruitment)		and normal tissue samples, suggesting that HPV infection can promote
	*HPV+: 88-HPV−: 288*	HOTAIR, PROM1, CCAT1, MUC19	HPV− > HPV+	MDSC aggregation into HNSCC areas. 2. There was an association between
	Normal/dysplasia: 30/30			the clinical stage and pathological grade and MDSC recruitment.

Abbreviations: CCL = Chemokine (C-C motif) ligand; C/N/LD/SD = carcinoma/normal tissue/low dysplasia/severe dysplasia; CXCL = Chemokine (C-X-C motif) ligand; HPV = human papilloma virus; IFN = interferon; MDSC = myeloid-derived suppressor cells; OPSCC = oropharyngeal SCC; OS = overall survival; LN/NM = lymph node/normal node; IL = interleukin; PD-1 = programmed cell death 1; PD-L1 = Programmed death ligand 1; PFS = progression-free survival; RFS = recurrence-free survival; (HN)SCC = (Head & Neck) squamous cell carcinoma; Tim-3 = T cell immunoglobulin and mucin-containing domain-3; Th = TNF-a; Necr = progression-free survival.

**Table 7:** Summary of evidence and findings.

Findings	Supporting Studies	Non-Supporting Studies
Confirmed		
1. The number of peripheral blood immune cells is higher in patients with SCC than in healthy individuals.	Al-Taei, 2013; Parikh, 2014; Ma, 2017; Lechner, 2017	-
2. CD8+ T cell infiltration is higher in HPV+ than in HPV− OP & HNSCC.	Nasman, 2012; Jung, 2012; Badoual, 2012;	Wansom, 2011; Balermpas, 2014
	Russel, 2013; Ritta, 2013; Nordfors, 2013; Krupar, 2014;	
	Oguejifor, 2015; Partlova, 2015; Kim, 2016;	
	Nguyen, 2016; Balermpas, 2016; Van Kempen, 2016;	
	Sivars, 2017; Poropatich, 2017	
3. High infiltration of CD8+ T cells is associated with better OS and RFS in HPV+ OP & HNSCC.	Nasman, 2012; Jung, 2012; Nordfors, 2013;	-
	Ward, 2014; Oguejiofor, 2015; Balermpas, 2016;	
	Poropatich, 2016; Nguyen, 2016; Kim, 2016,	
	Sivars, 2017	
4. CD8+ T cell infiltration is associated with the expression of checkpoint protein (PD-1) in HPV+ HNSCC.	Badoual, 2012; Partlova, 2015; Kim, 2016; Kansy, 2017	-
5. CD4+ T cell stromal infiltration is higher in HPV+ than in HPV− OPSCCs	Jung, 2012; Ritta, 2013; Nordfors, 2013; Krupar, 2014	Balermpas, 2014
6. HPV+ SCCs have higher intratumoral infiltration of Tregs than HPV− OP & HNSCCs.	Nasman, 2012; Badoual, 2012; Park, 2013; Ritta, 2013;	Wansom, 2011; Oguejiofor, 2015
	Krupar, 2014; Partlova, 2015; Nguyen, 2016;	Balermpas, 2014; Lechner, 2017;
	Punt, 2016; Kindt, 2017	Seminerio, 2019
7. A high infiltration of Tregs is associated with better OS or RFS in HPV+ oropharyngeal and HNSCCs.	Wansom, 2011; Nasman, 2012; Badoual, 2012;	Balermpas, 2014
	Park, 2013; Russel, 2013; Punt, 2016; Kindt, 2017;	
	Seminerio, 2019.	
Highly probable		
1. The number of peripheral blood CD8+ T cells (against E6-E7) is higher in patients with HPV+ SCC.	Parikh, 2014; Masterson, 2016;	Lukesova, 2014
2. HPV+ OPSCCs have a higher infiltration of CD68+ macrophages than HPV− OPSCCs.	Lyfor-Pike, 2013; Welters, 2017; Oguejiofor, 2017	Wansom, 2011
3. The level of MDSC infiltration was not associated with HPV status.	Russel, 2013; Yu, 2015; Ma, 2017	Partlova, 2015
Probable		
1. A high blood concentration of CD8+ T cells could enhance the immunoreactivity to antigen E7,	Masterson, 2016	-
which was associated with improved OS in patients with OPSCC		
2. Low CD8+ infiltration was associated with a high risk of metastases in HNSCC.	Balermpas, 2016	-
3. High CD8+ infiltration was responsible for high expression of CCL-17, CCL-21, IL-2, IL-4, IL-8,	Russel, 2013; Partlova, 2015	-
IL-10, IL-12, IL-17, IL-21, TNF-a; IFN-g.		
4. HPV− HNSCC have higher M2 infiltration than HPV+ SCC.	Ou, 2018	-
5. Patients with HPV− SCC and low M2 infiltration could have better 5-year PFS.	Ou, 2018	-
Controversial		
1. LC infiltration is influenced by HPV status.	Kindt et al., 2017	Perreira et al., 2011

Abbreviations: CCL = Chemokine (C-C motif) ligand; (HN)SCC = head & neck squamous cell carcinoma; HPV = human papilloma virus; IFN = interferon; IL = interleukin; LC = Langerhans cells; M2 = macrophage type 2; MDSC = myeloid-derived suppressor cell; OP = oropharyngeal; OS = overall survival; PD-1 = programmed death-1; PFS = progression-free survival; RFS = recurrence-free survival; TNF = tumor necrosis factor; Treg = T regulatory cells.
